# Supplementary material for: Differential Expression of miRNAs Involved in Response to Candidatus Liberibacter asiaticus Infection in Mexican Lime at Early and Late Stages of Huanglongbing Disease
Source: Plants (Basel). 2023 Feb 24;12(5):1039. doi: 10.3390/plants12051039 (PMC10005081; doi:10.3390/plants12051039)
Supplement: Supplementary file 1 [file plants-12-01039-s001.zip › Table S1.pdf]

Table S1. Criteria evaluated by ShortStack for miRNA analysis.

	Description
N0	Not analyzed due to run in --nohp mode
N1	No reads at all aligned in locus
N2	DicerCall was invalid (< 80% of reads in the Dicer size range defined by --dicermin and --dicermax)
N3	Major RNA abundance was less than 2 reads.
N4	Major RNA length is not in the Dicer size range defined by --dicermin and --dicermax
N5	Locus size is > than maximum allowed for RNA folding per option --foldsize (default is 300 nts)
N6	Locus is not stranded (>20% and <80% of reads aligned to top strand)
N7	RNA folding attempt failed at locus (if occurs, possible bug?)
N8	Strand of possible mature miRNA is opposite to that of the locus
N9	Retrieval of possible mature miRNA position failed (if occurs, possible bug?)
N10	General failure to compute miRNA-star position (if occurs, possible bug?)
N11	Possible mature miRNA had > 5 unpaired bases in predicted precursor secondary structure
N12	Possible mature miRNA was not contained in a single predicted hairpin
N13	Possible miRNA/miRNA* duplex had >2 bulges and/or >3 bulged nts
N14	Imprecise processing: Reads for possible miRNA, miRNA-star, and their 3p variants added up to less than 50% of the total reads at the locus
N15	Maybe. Passed all tests EXCEPT that the miRNA-star was not sequenced. INSUFFICIENT evidence to support a de novo annotation of a new miRNA family
Y	Yes. Passed all tests INCLUDING sequencing of the exact miRNA-star. Can support a de novo annotation of a new miRNA family.
